# Supplementary material for: Influence of Lactiplantibacillus plantarum and Saccharomyces cerevisiae Individual and Collaborative Inoculation on Flavor Characteristics of Rose Fermented Beverage
Source: Foods. 2025 May 24;14(11):1868. doi: 10.3390/foods14111868 (PMC12154399; doi:10.3390/foods14111868)
Supplement: Supplementary file 1 [file foods-14-01868-s001.zip › foods-3563902-supplementary.pdf]

Table S1 Taste threshold of amino acids and organic acids in rose fermented beverages

Components	Taste threshold (mg/100 mL)	Content (mg/100 mL)				TAVs			
		UFR	LFR	SFR	LSFR	UFR	LFR	SFR	LSFR
Sweet amino acids									
Thr	260[82]	0.395±0.011^{c}	3.425±0.141^{a}	0.395±0.068^{c}	3.171±0.06^{b}	0.002	0.013	0.002	0.012
Ser	150[82]	0.546±0.002^{c}	5.046±0.083^{a}	0.569±0.108^{c}	4.673±0.225^{b}	0.004	0.034	0.004	0.031
Gly	130[82]	0.617±0.068^{b}	32.000±1.060a	0.663±0.131^{b}	29.402±1.382^{a}	0.005	0.246	0.005	0.226
Ala	60[82]	2.235±0.072^{b}	16.544±0.113^{a}	1.493±0.105^{c}	15.367±0.901^{a}	0.037	0.276	0.025	0.256
Cys	2[83]	0.108±0.036^{c}	1.659±0.646^{ac}	0.494±0.199^{b}	0.564±0.206^{ab}	0.054	0.829	0.247	0.282
Met	30[82]	0.096±0.113^{b}	0.716±0.098^{a}	0.571±0.350^{ab}	0.548±0.133^{a}	0.003	0.024	0.019	0.018
Pro	300[82]	0.867±0.017^{b}	15.099±1.181^{a}	0.128±0.215^{c}	1.146±1.962^{bc}	0.003	0.050	0.000	0.004
Bitter amino acids									
Val	40[82]	0.418±0.036^{c}	4.799±0.259^{a}	1.244±0.119^{b}	4.59±0.139^{a}	0.010	0.120	0.031	0.115
Ile	90[82]	0.337±0.038^{b}	3.108±0.252^{a}	0.478±0.039^{b}	2.45±0.196^{a}	0.004	0.035	0.005	0.027
Lue	190[82]	0.523±0.013^{b}	6.770±0.874^{a}	0.759±0.118^{b}	4.926±0.062^{a}	0.003	0.036	0.004	0.026
Tyr	260[83]	0.357±0.012^{b}	1.533±0.573^{ab}	0.235±0.078^{b}	1.030±0.031^{a}	0.001	0.006	0.001	0.004
Phe	90[82]	0.767±0.027^{b}	3.900±1.072^{a}	0.247±0.052^{c}	2.961±0.087^{a}	0.009	0.043	0.003	0.033
His	20[82]	0.073±0.063^{a}	0.935±0.756^{a}	0.162±0.093^{a}	0.155±0.017^{a}	0.004	0.047	0.008	0.008
Arg	50[82]	0.538±0.039^{b}	12.194±1.521^{a}	0±0^{c}	9.966±0.538^{a}	0.011	0.244	0.000	0.199
Trp	90[84]	0.648±0.004^{c}	18.613±0.539^{a}	0.397±0.021^{d}	13.154±1.444^{b}	0.007	0.207	0.004	0.146
Umami amino acids									
Asp	100[82]	10.349±0.490^{b}	19.065±1.128^{a}	6.226±0.068^{c}	15.978±0.418^{a}	0.103	0.191	0.062	0.160
Glu	30[82]	2.124±0.100^{b}	17.432±0.776^{a}	2.466±0.278^{b}	15.443±0.848^{a}	0.071	0.581	0.082	0.515
Organic acids									
Tartaric acid	1.5[82]	20.04±1.52^{d}	37.884±0.374^{b}	29.274±0.036^{c}	47.704±2.654^{a}	13.360	25.256	19.516	31.344
Malic acid	50[82]	72.595±7^{ab}	77.955±0.926^{a}	78.551±0.1^{a}	65.083±1.843^{b}	1.452	1.559	1.571	1.302
Lactic acid	126[82]	0±0^{c}	475.194±4.23^{a}	0±0^{c}	329.36±5.911^{b}	0.000	3.771	0.000	2.614
Acetic acid	10.6[82]	0±0^{d}	97.979±2.868^{a}	20.938±0.795^{c}	68.764±3.22^{b}	0.000	9.243	1.975	6.487
Citric acid	45[82]	0±0^{d}	55.628±0.326^{a}	13.556±7.574^{c}	46.44±0.594^{b}	0.000	1.236	0.301	1.032
Succinic acid	10.6[82]	69.156±5.907^{c}	78.321±0.254^{ac}	75.645±0.26^{bc}	72.443±4.784^{c}	6.524	7.389	7.136	6.834

Note: Taste threshold value (mg/100 mL) in water

Table S2 Volatile compounds present in rose beverages were identified using HS-SPME/GC–MS.

Compounds	Average RI	Quant mass	Relative content (ug/mL)			
			LSFR	LFR	SFR	UFR
**Alcohols**						
(1R,6R)-3-Methyl-6-(1-Methylethenyl)-2-Cyclohexen-1-Ol	1182.17	84.02	0.59±0.09^{a}	0.15±0.02^{c}	0.02±0.04^{d}	0.3±0.01^{b}

(1aS,4aS,7R,7aS,7bS)-1,1,7-Trimethyl-4-Methylidene-1a,2,3,4a,5,6,7a,7b-Octahydrocyclopropa[h]azulen-7-Ol	1579.48	91.02	0.17±0.01^{a}	0.16±0^{b}	0±0^{c}	0±0^{c}
(1r,2r,5s)-5-Methyl-2-(Prop-1-En-2-Yl)Cyclohexanol	1149.35	71.02	0.25±0.03^{a}	0.13±0.01^{b}	0±0^{c}	0.12±0^{b}
(1s,3as,4s,5s,7ar,8r)-5-Isopropyl-1,7a-Dimethyloctahydro-1h-1,4-Methanoinden-8-Ol	1612.21	95.05	1.21±0.1^{b}	1.47±0.14^{a}	0.11±0.01^{d}	0.55±0.03^{c}
10-epi-.α.-Cadinol	1644.09	161.09	3.23±0.51^{c}	4.33±0.16^{a}	0.84±0.04^{d}	3.79±0.22^{b}
(E)-3,7,11-Trimethyl-1,6,10-Dodecatrien-3-Ol	1561.06	69.05	0.6±0.02^{b}	0.73±0.04^{b}	3.59±0.77^{a}	0.22±0.02^{b}
1,7,7-Trimethylbicyclo[2.2.1]Heptan-2-Ol	1173.00	95.05	1.85±0.04^{b}	3.24±0.04^{a}	0.12±0.01^{d}	1.27±0.07^{c}
α,2,6,6-Tetramethyl-1-Cyclohexene-1-Propanol	1443.19	123.08	3.4±0.19^{b}	4.55±0.13^{a}	0.2±0.01^{d}	1.82±0.05^{c}
Decan-1-Ol	1273.48	55.03	2.45±0.27^{a}	0.88±0.02^{b}	0.24±0.02^{c}	0±0^{d}
[1ar-(1aα,4β,4aβ,7α,7aβ,7bα)]-1,1,4,7-Tetramethyldecahydro-1h-Cyclopropa[E]Azulen-4-Ol	1588.69	185.08	0.12±0.01^{b}	0.16±0.01^{a}	0±0^{c}	0±0^{c}
2-Propylheptan-1-Ol	1212.52	43.04	0.78±0.1^{b}	1.05±0.01^{a}	0±0^{d}	0.52±0.01^{c}
Hexan-1-Ol	866.76	56.05	1.31±0.2^{b}	2.31±0.2^{a}	0.12±0.01^{c}	0.18±0.01^{c}
Nonan-1-Ol	1172.73	56.05	3.43±0.29^{b}	5.19±0.09^{a}	0±0^{d}	0.26±0.02^{c}
Octan-1-Ol	1072.47	56.04	0.99±0.12^{a}	0.98±0.05^{a}	0.11±0.07^{c}	0.51±0.03^{b}
2-((2s,4ar)-4a,8-Dimethyl-1,2,3,4,4a,5,6,7-Octahydronaphthalen-2-Yl)Propan-2-Ol	1625.88	189.10	0.6±0.02^{b}	0.97±0.01^{a}	0.27±0.02^{c}	0.23±0.03^{d}
2,6,6-Trimethyl-2-Cyclohexen-1-Methanol	1176.99	123.06	0.29±0.03^{a}	0.31±0.01^{a}	0±0^{c}	0.19±0^{b}
Dodecan-2-Ol	1404.00	45.01	0.34±0.04^{a}	0.28±0.02^{a}	0.09±0.01^{a}	0.25±0.33^{a}
Cis-5-Ethenyltetrahydro-α,α,5-Trimethyl-2-Furanmethanol	1070.17	59.02	0.55±0.15^{b}	0.77±0.02^{a}	0.22±0.01^{d}	0.38±0.01^{c}
(2S,5R)-5-(4-Methyl-3-Cyclohexen-1-yl)-2,2,5-Trimethyltetrahydrofuran-3-ylmethanol	1657.28	143.06	0.83±0.06^{b}	1.04±0.05^{a}	0.09±0^{c}	0.12±0^{c}
(2R,2α,4aα,8aβ)-1,2,3,4,4a,5,6,7-Octahydro-α,α,4a,8-Tetramethylnaphthalen-2-ylmethanol	1634.61	189.13	24.47±1.1^{b}	50.37±1.07^{a}	8.38±0.68^{c}	4.31±0.25^{d}
(2R,2α,4aα,8aα)-1,2,3,4,4a,8a-Hexahydro-α,α,4a,8-Tetramethylnaphthalen-2-ylmethanol	1633.11	202.13	0.46±0.11^{b}	0.72±0.03^{a}	0±0^{d}	0.18±0.01^{c}
[2r-(2α,4aα,8aβ)]-Decahydro-α,α,4a-Trimethyl-8-Methylenenaphthalen-2-ylmethanol	1658.47	59.03	31.04±0.9^{b}	41.57±1.14^{a}	3.28±0.21^{d}	9.16±0.48^{c}
Nonan-2-Ol	1102.23	45.02	4.82±0.41^{b}	9.66±0.26^{a}	0.38±0.04^{c}	1.06±0.03^{c}
(E)-2-Nonen-1-Ol	1169.17	57.02	0±0^{c}	0.25±0.03^{a}	0±0^{c}	0.08±0^{b}
Tetradecan-2-Ol	1605.56	45.00	0.09±0^{c}	0.13±0.01^{b}	0.21±0.03^{a}	0±0^{d}
Undecan-2-Ol	1302.69	45.03	11.57±0.61^{b}	21.06±1.69^{a}	1.07±0.02^{c}	0.1±0.01^{c}

(Z)-3,7-Dimethyl-3,6-Octadien-1-Ol	1264.90	67.02	1.43±0.1[a]	1.5±0.02[a]	0±0[b]	0±0[b]
(R)-4-Methyl-1-(1-Methylethyl)-3-Cyclohexen-1-Ol	1181.24	71.02	1.47±0.11[c]	2.58±0.05[b]	0.19±0.01[d]	4.01±0.19[a]
(E)-3-Hexen-1-Ol	852.18	67.01	0.18±0.02[b]	0.32±0.04[a]	0±0[c]	0±0[c]
5-Methyl-2-(1-Methylethenyl)-4-Hexen-1-Ol	1164.28	69.04	0.26±0.05[c]	1.26±0.04[b]	0.19±0.01[c]	2.3±0.11[a]
3,7,11-Trimethyl-6,10-Dodecadien-1-Ol	1688.89	69.04	0±0[b]	0±0[b]	1.84±0.13[a]	0±0[b]
7-Methyl-3-Methylene-6-Octen-1-Ol	1217.34	69.05	4.26±0.17[a]	4.28±0.07[a]	0.15±0.01[c]	2.53±0.11[b]
(S)-3,7-Dimethyl-7-Octen-1-Ol	1215.62	55.04	0.49±0.02[c]	4.33±0.05[a]	0.14±0.01[d]	3.03±0.06[b]
2,6-Dimethyl-7-Octene-2,6-Diol	1086.85	71.02	0.3±0.03[b]	0.49±0.01[a]	0±0[c]	0.27±0.01[b]
9-Decen-2-Ol	1486.37	67.03	0.87±0.08[b]	1.15±0.09[a]	0.15±0.02[c]	0.11±0[c]
Benzyl Alcohol	1038.44	79.03	9.52±2.28[b]	15.57±1.42[a]	0±0[c]	0±0[c]
2-Methyl-5-(1-Methylethenyl)-2-Cyclohexen-1-Ol	1002.27	119.05	0.31±0.05[b]	0.28±0.01[b]	0±0[c]	0.56±0.02[a]
3,7-Dimethyl-6-Octen-1-Ol	1228.07	69.05	149.21±11.13[b]	249.46±11.5[a]	8.68±0.51[d]	116.72±5.15[c]
(R)-3,7-Dimethyl-6-octen-1-Ol, 1-(Trimethylsilyl) Derivative	1309.61	177.07	0.14±0.02[a]	0.15±0[a]	0±0[b]	0±0[b]
2-Methyl-5-(1-Methylethenyl)-Cyclohexanol	1029.39	57.05	2.49±0.09[a]	0.74±0.04[b]	0.2±0.03[c]	0.82±0.1[b]
2-Methyl-2-(4-Methyl-3-Pentenyl)-Cyclopropanemethanol	1320.61	69.04	7.7±0.73[a]	5.15±0.31[b]	0.31±0.03[d]	1.89±0.21[c]
(R)-3,7-Dimethyl-6-Octen-1-Ol	1228.28	41.05	188.76±5.38[b]	236.11±4.87[a]	9.36±0.56[d]	127.9±5.28[c]
Di-Epi-1,10-Cubenol	1628.97	161.09	0.18±0.03[a]	0.14±0.01[b]	0.17±0.02[a]	0.08±0[c]
(E)-3,7-Dimethylocta-2,6-Dien-1-Ol	1251.71	69.06	100.92±3.03[b]	112.6±8.41[a]	0.44±0.06[d]	62.68±5.69[c]
(1R,2S,5R)-5-Methyl-2-(1-Methoxyethyl)Cyclohexanol	1177.95	123.06	0.25±0.05[b]	0.33±0.01[a]	0±0[d]	0.18±0[c]
3,7-Dimethylocta-1,6-Dien-3-Ol	1099.18	71.03	26.08±2.79[b]	54.49±1.5[a]	1.77±0.12[c]	27.57±1.4[b]
Phenylethyl Alcohol	1114.60	91.04	115.18±37.77[b]	226.32±11.13[a]	126.49±16.82[b]	22.48±2.3[c]
(E)-3-Caren-2-Ol	1055.31	119.03	0.5±0.03[b]	0.49±0.01[b]	0.16±0.01[c]	0.53±0.01[a]
α,α,6,8-Tetramethyl-Tricyclo[4.4.0.0(2,7)]Dec-8-Ene-3-Methanol	1674.43	59.02	0.56±0.13[a]	0.63±0.02[a]	0.11±0.01[c]	0.31±0.01[b]
α-Epi-7-Epi-5-Eudesmol	1609.08	59.02	0.89±0.05[b]	1.46±0.03[a]	0.22±0.01[d]	0.28±0.02[c]
α-Terpineol	1195.88	59.03	15.43±2.09[b]	18.79±0.48[a]	3.29±0.11[d]	7.8±0.11[c]
(2Z)-3,7-Dimethylocta-2,6-Dien-1-Ol	1217.52	133.03	14±4.51[a]	9.4±0.41[b]	0.23±0.05[c]	2.77±0.16[c]
**Esters**						
(Z)-3,7-Dimethyl-2,6-Octadienylethanoate	1357.28	69.05	3.3±2.75[a]	0±0[b]	0±0[b]	0.2±0.1[b]
Bis(2-Methylpropyl) Benzene-1,2-Dicarboxylate	1860.47	148.97	0±0[b]	0±0[b]	0.78±0.05[a]	0±0[b]
(E)-Methyl 1-Methoxy-5-Methyl-1,5-Hexadiene-3-Carboxylate	1435.50	129.03	0.88±0.03[a]	0.82±0.01[b]	0.12±0.01[d]	0.67±0.01[c]
2,2,4-Trimethyl-1,3-Pentanediol Diisobutyrate	1586.10	71.02	2.2±0.06[b]	2.5±0.08[a]	1.25±0.03[d]	1.9±0.02[c]
2-Phenylethyl Acetate	1254.48	104.03	36.15±19.88[a]	10.48±0.93[b]	5.79±0.17[b]	7.28±0.96[b]
Ethyl 3,7-Dimethyl-Oct-6-Enoate	1327.83	69.06	0.6±0.26[a]	0±0[c]	0.23±0.01[b]	0±0[c]

2-Isopropenyl-5-Methyl-4-Hexen-1-yl Acetate	1281.57	69.04	2.89±0.13[c]	5.27±0.12[a]	0.38±0.01[d]	4.02±0.12[b]
3,7-Dimethyl-6-Octen-1-yl Acetate	1348.67	43.02	1.8±0.44[a]	1.1±0.63[b]	1.13±0.2[b]	0.5±0.04[b]
Phenylmethyl Acetate	1162.92	108.02	1.44±0.13[a]	0.65±0.02[b]	0±0[d]	0.45±0.01[c]
Undec-2-Enyl Acetate	1105.12	109.02	0.11±0.01[b]	0.22±0.02[a]	0±0[d]	0.08±0[c]
Ethyl Benzeneacetate	1242.46	91.02	0.24±0.03[d]	0.32±0.01[c]	2.14±0.03[a]	0.38±0.01[b]
Ethyl Benzoate	1171.23	104.99	0.44±0.1[ab]	0.48±0.01[a]	0.37±0.03[c]	0.44±0.04[ab]
Diethyl Butanedioate	1178.92	100.99	0±0[b]	0±0[b]	2.42±0.12[a]	0±0[b]
Ethyl 2-Methylbutanoate	841.12	57.05	0±0[b]	0±0[b]	0.47±0.02[a]	0±0[b]
Ethyl 3-Methylbutanoate	846.26	88.01	0±0[b]	0±0[a]	0.3±0.01[a]	0±0[b]
Ethyl 9-Hexadecenoate	1970.18	55.03	0±0[b]	0±0[b]	3.81±0.34[a]	0±0[b]
Ethyl Octanoate	1195.31	88.03	0.94±0.17[b]	0.24±0.02[c]	48.99±2.1[a]	0.19±0.01[c]
Ethyl Hexanoate	996.88	88.02	0.15±0.01[b]	0±0[c]	11±0.35[a]	0±0[c]
3,7-Dimethyl-1,6-Octadien-3-yl Acetate	1376.99	43.02	0.92±0.37[b]	1.34±0.24[a]	0±0[c]	1.15±0.07[b]
3-Methylbutyl Acetate	871.09	43.02	2.27±0.4[b]	0.31±0.03[c]	16.85±0.59[a]	0.3±0.01[c]
3-Methylbutyl Dodecanoate	1844.11	70.05	0±0[b]	0±0[b]	0.77±0.15[a]	0±0[b]
2-(Methylamino)Benzoic Acid Methyl Ester	1407.57	165.03	4.56±0.98[b]	10.57±0.23[a]	4.04±0.2[b]	10.09±0.17[a]
Methyl 2-Hydroxybenzoat	1192.58	119.98	4.07±0.51[a]	4.45±0.1[a]	0.12±0.02[c]	0.76±0.03[b]
Methyl Tetradecanoate	1724.74	73.99	0.1±0.01[b]	0.12±0.01[a]	0.08±0.01[c]	0.08±0[c]
Methyl Octanoate	1122.58	74.01	0.32±0.01[b]	0.3±0.01[c]	0.28±0.01[d]	0.34±0.01[a]
Ethyl 2-hydroxy-4-Methylpentanoate	1057.21	69.04	0±0[c]	0.25±0.08[b]	0.34±0.05[a]	0±0[c]
(3,7-Dimethyl-6-octenyl) 4-Methylpentanoate	1228.21	81.06	106.41±2.55[b]	127.03±2.86[a]	4.61±0.27[d]	63.12±2.99[c]
**Acids**						
3,7-Dimethyl-6-Octenoic Acid	1320.28	69.04	8.63±0.49[a]	5.34±0.32[b]	0.16±0.02[d]	1.68±0.23[c]
(E)-3,7-Dimethyl-2,6-Octadienoic Acid	1371.68	69.05	64.25±5.16[a]	66.97±4.06[a]	0±0[b]	0±0[b]
2-Methylbutanoic Acid	876.73	74.01	0±0[b]	0±0[b]	0.27±0.09[a]	0±0[b]
Hexanoic Acid	1009.87	60.00	0.14±0.02[bc]	0.22±0.11[b]	0.51±0.24[a]	0±0[c]
Decanoic Acid	1371.68	69.05	24.47±5.07[a]	0.18±0[b]	27.67±2.59[a]	0±0[c]
Nonanoic Acid	1281.50	45.02	4.65±0.17[b]	7.5±0.42[a]	0±0[d]	0.42±0.01[c]
Octanoic Acid	1193.32	60.00	16.14±0.8[a]	0.54±0.03[b]	16.18±1.57[a]	0.08±0.01[b]
**Phenols**						
(1R,4aS,8aR)-1,4a-Dimethyl-7-(1-Methylethylidene)Decahydro-1-Naphthalenol	1660.98	157.05	0.57±0.14[a]	0.59±0.02[a]	0.11±0.01[c]	0.28±0[b]
2,4-Bis(1,1-Dimethylethyl)Phenol	1505.49	191.11	43.1±2.46[b]	68.67±16.16[a]	7.9±0.25[d]	25.54±1.28[c]
2,5,5-Trimethyl-1,2,3,4,4a,5,6,7-Octahydro-2-Naphthalenol	1404.36	121.06	0.4±0.04[a]	0.21±0.01[b]	0±0[c]	0±0[c]
4-Allyl-2-Methoxyphenol	1353.61	164.05	117.38±11.09[b]	193.53±5.83[a]	2.02±0.1[d]	31.42±0.56[c]
4-Allyl-1,2-Dimethoxybenzene	1399.29	178.07	49.99±4.47[b]	59.37±1.64[a]	6.78±0.36[c]	60.95±1.43[a]
(Z)-2-Methoxy-4-(1-Propenyl)Phenol	1452.14	164.03	0.64±0.08[b]	0.75±0.02[a]	0±0[c]	0±0[c]
2-Methoxy-6-(2-Propenyl)Phenol	1356.38	164.01	0±0[b]	157.67±5.9[a]	2.85±0.22[b]	0.81±0.06[b]
5-Isopropyl-2-Methylphenol	1302.77	135.04	1.1±0.39[c]	4.12±0.19[a]	0.82±0.05[c]	2.19±0.09[b]
**Aldehydes**						

2,5-Dihydroxybenzaldehyde, 2-(trimethylsilyl) Derivative	1098.01	266.96	2.6±0.14[ab]	2.38±0.22[b]	1.61±0.29[c]	2.88±0.17[a]
(Z)-3,7-Dimethyl-2,6-Octadienal	1238.66	41.04	1.23±0.11[b]	1.43±0.17[b]	0.14±0.02[c]	8.11±0.42[a]
(E)-2-Hexenal	848.29	41.03	0±0[c]	0±0[c]	0.15±0.04[b]	0.4±0.04[a]
(E)-2-Nonenal	1161.39	41.03	0.14±0.01[b]	0.09±0.01[c]	0±0[d]	0.36±0.02[a]
(E)-2-Octenal	1059.04	69.98	0±0[c]	0.09±0[b]	0±0[c]	0.23±0.01[a]
3-Furaldehyde	829.62	95.99	0±0[c]	0.68±0.05[b]	0.85±0.15[b]	1.85±0.16[a]
5-Isopropenyl-2-Methylcyclopent-1-Enecarboxaldehyde	1213.26	107.03	0.12±0.04[b]	0.2±0[c]	0±0[d]	0.33±0.01[a]
Benzaldehyde	961.04	106.01	0.61±0.06[bc]	1.03±0.14[b]	0.17±0.03[c]	5.67±0.73[a]
2,4,5-Trimethyl-Benzaldehyde	1303.85	147.03	0.4±0.08[b]	0.64±0.24[a]	0.09±0.01[c]	0.3±0.04[b]
2,4-Dimethyl-Benzaldehyde	1217.52	133.03	33.8±2.93[a]	40.98±9.15[a]	1.8±0.49[b]	8.9±0.76[b]
3,4-Dimethyl-Benzaldehyde	1223.64	133.03	0±0[d]	0.09±0.01[c]	0.5±0.07[a]	0.19±0.05[b]
4-Propyl-Benzaldehyde	1276.96	91.03	0.34±0.01[b]	6.69±0.88[a]	0±0[b]	0.21±0.02[b]
Benzeneacetaldehyde	1046.70	120.02	0.33±0.07[c]	0.46±0.01[b]	0±0[d]	1.54±0.06[a]
α-Ethylidenebenzeneacetaldehyde	1270.00	115.01	0.17±0.01[b]	0.48±0.03[a]	0±0[c]	0.48±0.01[a]
β-Methyl-Benzenepropanal	1197.17	148.04	0.22±0.01[b]	0.25±0[a]	0±0[d]	0.21±0[c]
Decanal	1205.91	57.03	0.38±0.06[c]	0.46±0.02[b]	0.17±0[d]	0.58±0.02[a]
Furfural	828.68	95.99	0±0[d]	0.84±0.1[b]	0.46±0.11[c]	1.89±0.28[a]
Heptanal	900.22	45.02	0.74±0.04[b]	1.43±0.1[a]	0±0[c]	0±0[c]
Nonanal	1103.96	57.04	1.42±0.11[b]	1.39±0.08[b]	0.33±0.02[c]	6.17±0.23[a]
Octanal	1002.49	67.02	0.28±0.02[c]	0.44±0.02[a]	0.08±0[d]	0.38±0.01[b]
2-Methyl-3-Phenyl-Propanal	1244.11	73.01	0.31±0.03[b]	0.34±0.01[a]	0.25±0.02[c]	0.26±0.01[c]
**Ketones**						
1b,5,5,6a-Tetramethyl-Octahydro-1-Oxa-Cyclopropa[A]Inden-6-One	1185.57	77.00	0.32±0.03[b]	0.42±0.02[a]	0.11±0.02[d]	0.2±0.01[c]
5-Heptyldihydro-2(3h)-Furanone	1363.34	85.00	2.32±0.92[a]	1.23±0.03[b]	0.7±0.19[b]	0.69±0.18[b]
5-Hexyldihydro-2(3h)-Furanone	1468.50	85.00	0.32±0.01[b]	1.21±0.03[a]	0.17±0[c]	0.19±0.04[c]
2,6-Bis(1,1-Dimethylethyl)-2,5-Cyclohexadiene-1,4-Dione	1460.97	177.07	0.21±0.01[a]	0.2±0.02[a]	0±0[c]	0.15±0[b]
4-(2,6,6-Trimethyl-1,3-Cyclohexadien-1-Yl)-2-Butanone	1429.91	119.04	1.36±0.13[a]	1.08±0.05[b]	0.08±0[c]	0.1±0[c]
4-(2,6,6-Trimethyl-1-Cyclohexen-1-Yl)-2-Butanone	1433.24	121.05	0.33±0.01[b]	0.42±0.03[a]	0±0[d]	0.16±0[c]
1-(2,6,6-Trimethyl-1,3-Cyclohexadien-1-Yl)-2-Buten-1-One	1379.01	69.02	13.56±1.76[a]	14.17±0.23[a]	7.58±0.22[b]	5.64±0.23[c]
4-(1-Methylethyl)-2-Cyclohexen-1-One	1188.49	96.02	0±0[c]	0.4±0.01[b]	0±0[c]	0.54±0.01[a]
Heptan-2-One	885.29	58.00	0.24±0.03[a]	0.16±0.04[b]	0±0[d]	0.08±0[c]
Nonan-2-One	1090.16	58.02	1.15±0.12[a]	0.86±0.18[b]	0.4±0.03[c]	0.17±0[d]
Undecan-2-One	1292.23	58.02	0.44±0.01[a]	0.36±0.04[b]	0.38±0.01[b]	0.13±0[c]
3,5-Octadien-2-One	1071.33	95.01	0.14±0.01[c]	0.35±0.02[a]	0±0[d]	0.29±0.03[b]
4-(2,6,6-Trimethylcyclohexa-1,3-Dienyl)But-3-En-2-One	1428.05	43.02	0.3±0.02[b]	1.06±0.03[a]	0.17±0.01[c]	0.15±0.01[c]
6,10-Dimethyl-5,9-Undecadien-2-One	1447.19	43.02	0.38±0.02[b]	0.37±0.03[b]	0.42±0.01[b]	1.64±0.06[a]

6-Methyl-5-Hepten-2-One	982.59	43.01	0.1±0.01^{b}	0±0^{c}	0.1±0.02^{b}	0.66±0.04^{a}
1-Phenylethanone	1067.87	104.99	0.26±0.02^{b}	0.24±0.03^{b}	0±0^{c}	0.38±0.02^{a}
3,5,5-Trimethylcyclohex-2-En-1-One	1122.02	82.01	5.84±0.81^{a}	3.9±0.09^{b}	0.31±0.01^{c}	3.82±0.14^{b}
(E)-β-Ionone	1479.32	177.08	0±0^{d}	0.2±0.01^{b}	0.17±0.01^{c}	2.02±0.01^{a}
4-(2,6,6-Trimethylcyclohexa-1,3-Dien-1-yl)-3-Buten-2-One	1422.07	121.01	0±0^{c}	0.08±0.01^{b}	0±0^{c}	0.13±0.01^{a}
**Benzene**						
1-(1,5-Dimethyl-4-Hexenyl)-4-Methylbenzene	1480.94	132.04	0.19±0^{b}	0.17±0^{bc}	0.99±0.06^{a}	0.14±0.01^{c}
1,2,3,5-Tetramethylbenzene	1149.05	119.04	1.49±0.06^{b}	1.63±0.05^{a}	0.69±0.03^{c}	1.64±0.06^{a}
1,2,3-Trimethoxy-5-(2-Propenyl)-Benzene	1546.86	208.07	0.51±0.07^{b}	0.64±0.02^{a}	0.08±0.01^{c}	0.48±0.04^{b}
1,2,3-Trimethylbenzene	1017.69	105.03	0.54±0.02^{b}	0.54±0.03^{b}	0.18±0.03^{c}	0.65±0.04^{a}
1,2,4,5-Tetramethylbenzene	1117.56	119.05	2.79±0.27^{c}	3.14±0.08^{b}	1.44±0.08^{d}	3.52±0.29^{a}
1,2-Diethylbenzene	1120.78	119.04	0.49±0.03^{a}	0.39±0.02^{b}	0±0^{d}	0.36±0.01^{c}
1,3,5-Trimethoxybenzene	1409.35	168.04	14.08±2.74^{ab}	16.03±0.13^{a}	2.73±0.17^{c}	12.5±0.4^{b}
1-Ethyl-2-methylbenzene	973.90	105.00	0.16±0.02^{a}	0.16±0.01^{a}	0±0b	0±0^{b}
1-Methyl-3-(1-Methylethenyl)-Benzene	1089.25	132.05	2.15±0.38^{b}	1.8±0.05^{c}	0.38±0.02^{d}	2.65±0.1^{a}
1-Methyl-3-(1-Methylethyl)-Benzene	1022.33	119.05	6.63±0.74^{a}	4.5±0.22^{b}	0.36±0.01^{c}	7.1±0.19^{a}
1-Methyl-4-(1-Methylethenyl)-Benzene	1089.09	132.05	2.2±0.39^{b}	1.9±0.06^{b}	0.42±0.01^{c}	2.78±0.05^{a}
2-Ethyl-1,4-Dimethyl-Benzene	1082.06	119.04	0.75±0.02^{b}	0.74±0.01^{b}	0.25±0.03^{c}	0.81±0.02^{a}
Hexamethyl-Dewar Benzene	1652.07	59.02	1.6±0.18^{b}	2.15±0.03^{a}	0.22±0.01^{d}	0.54±0.02^{c}
1,3,5-Trimethylbenzene	990.45	105.03	0.26±0.01^{c}	0.29±0.01^{b}	0.17±0.02^{c}	0.32±0.02^{a}
1,2-Dimethylbenzene	862.06	91.01	0.19±0.01^{c}	0.22±0.02^{a}	0.09±0^{d}	0.21±0.01^{b}
**Ethers**						
(2s,4r)-4-Methyl-2-(2-Methylprop-1-En-1-Yl)Tetrahydro-2h-Pyran	1108.81	139.07	7.08±0.52^{c}	25.41±1.05^{a}	0.6±0.03^{d}	10.1±0.38^{b}
3,6-Dihydro-4-Methyl-2-(2-Methyl-1-Propenyl)-2h-Pyran	1151.11	68.04	6.41±0.42^{b}	9.5±0.1^{a}	0.27±0.01^{d}	2.34±0.09^{c}
3,5-Dimethoxytoluene	1266.44	152.04	0.45±0.05^{c}	0.95±0.02^{b}	0±0^{d}	1.37±0.05^{a}
Methyl 9-Decen-1-Ol Ether	1486.29	81.04	1.27±0.02^{b}	1.85±0.11^{a}	0.68±0.04^{c}	0.33±0.01^{d}
2-Methoxy-2-methylbutane	1384.29	73.01	17.48±2.48^{b}	0.44±0.02^{c}	28.17±1.17^{a}	0.27±0.01^{c}
3,6-Dimethyl-2,3,3a,4,5,7a-Hexahydrobenzofuran	1186.30	137.04	0.52±0.0^{b}	0.85±0.01^{a}	0±0^{d}	0.11±0^{c}
(2E)-1-Ethoxy-3,7-Dimethylocta-2,6-Diene	1281.15	41.04	4.85±0.19^{b}	8.62±0.23^{a}	0.31±0.02^{d}	3.35±0.13^{c}
(3S,3aR,6R,8aS)-3-(Methoxymethyl)-7,7-Dimethyl-8-Methyleneoctahydro-1H-3a,6-Methanoazulene	1496.55	118.98	1.57±0.07^{b}	1.79±0.04^{a}	0.09±0.01^{c}	0±0^{d}
**Terpenes**						
4,7,7-Trimethylbicyclo[4.1.0]Hept-2-Ene	1237.32	121.06	1.29±0.3^{b}	2.38±0.08^{a}	1.06±0.04^{b}	0.43±0.08^{c}
(1S,4R,4aR)-1-Isopropyl-4-Methyl-7-Methylene-1,2,3,4,4a,5,6,7-Octahydronaphthalene	1513.11	73.00	0.12±0.01^{a}	0.14±0^{a}	0.13±0.03^{a}	0.1±0^{b}
(4ar,8as)-4a-Methyl-1-Methylene-7-(Propan-2-Ylidene)Decahydronaphthalene	1538.40	161.09	1.86±0.08^{a}	1.83±0.05^{a}	0.39±0.01^{c}	1.05±0.01^{b}

(E)-4,8-Dimethylnona-1,3,7-Triene	1111.57	69.03	0.34±0.09^{a}	0.26±0.03^{b}	0.02±0.04^{c}	0.08±0^{c}
(E)-3,7,11-Trimethyltrideca-1,6,10-Triene	1451.55	69.04	1.75±0.04^{b}	1.51±0.02^{c}	4.33±0.13^{a}	0.82±0.02^{d}
4-Methyl-1-(Propan-2-yl)Cyclohex-1-Ene	1056.39	93.04	3.09±0.35^{a}	2.39±0.07^{b}	0.11±0^{d}	2.08±0.1^{c}
(E)-5-Methyl-1,3,6-Heptatriene	1028.10	91.03	6.56±0.88^{a}	5.19±0.11^{b}	0.2±0.01^{d}	3.63±0.45^{c}
(Z)-3,7-Dimethyl-1,3,6-Octatriene	1044.88	93.04	19.66±1.47^{a}	17.73±0.7^{b}	0.28±0.01^{c}	16.32±1.05^{b}
2,7-Dimethyl-1,3,7-Octatriene	1014.73	81.04	0.78±0.06^{a}	0.56±0.04^{b}	0±0^{c}	0.54±0.03^{b}
1,5,5-Trimethyl-6-Methylene-Cyclohexene	1209.57	121.04	0.16±0.04^{b}	0.26±0.01^{a}	0.12±0.01^{c}	0±0^{4}
1,5,6,7-Tetramethylbicyclo[3.2.0]Hepta-2,6-Diene	1205.10	133.04	0.28±0.04^{b}	0.37±0.09^{a}	0.11±0^{c}	0.24±0.01^{b}
2-Methyl-6-Methylene-1,7-Octadiene	974.46	79.02	0.44±0.03^{a}	0.4±0.02^{b}	0±0^{c}	0.39±0.01^{b}
11-Tricosene	1491.09	83.03	0.28±0.02^{a}	0.25±0.01^{b}	0.12±0.01^{d}	0.15±0.01^{c}
[3r-(3α,3aβ,7β,8aα)]-Octahydro-3,8,8-Trimethyl-6-Methylene-1h-3a,7-Methanoazulene	1426.07	161.06	0.09±0.01^{c}	0.11±0^{b}	0.08±0.01^{d}	0.15±0^{a}
2,6,10,10-Tetramethyl-1-Oxaspiro[4.5]Dec-6-Ene	1315.08	138.06	1.43±0.37^{b}	1.75±0.03^{a}	0±0^{d}	0.29±0^{c}
2,6-Dimethyl-2,4,6-Octatriene	1127.10	121.05	0.72±0.03^{a}	0.6±0.01^{c}	0±0^{d}	0.65±0.01^{b}
(E,Z)-2,6-Dimethyl-2,4,6-Octatriene	1139.11	121.04	0.66±0.04^{a}	0.56±0.02^{b}	0±0^{c}	0.65±0.03^{a}
(E,E)-2,6-Dimethyl-1,3,5,7-Octatetraene	1129.43	91.02	1.44±0.07^{a}	1.33±0.06^{b}	0±0^{d}	1.06±0.05^{c}
4,7,7-Trimethylbicyclo[4.1.0]Hept-4-Ene	1014.22	121.06	3.85±0.49^{a}	2.89±0.11^{b}	0.1±0^{d}	2.37±0.15^{c}
2-Isopropenyl-4a,8-Dimethyl-1,2,3,4,4a,5,6,7-Octahydronaphthalene	1474.21	189.12	13.38±2.05^{b}	20.67±0.3^{a}	5.83±0.25^{d}	3.59±0.04^{c}
2-Methylbicyclo[4.3.0]Non-1(6)-Ene	1387.32	121.05	0.12±0.04^{a}	0±0^{b}	0±0^{b}	0±0^{b}
(3R,8aR)-5,8a-Dimethyl-3-(Prop-1-En-2-yl)-1,2,3,7,8,8a-Hexahydronaphthalene	1615.70	187.10	1.15±0.08^{b}	1.66±0.06^{a}	0.36±0.03^{c}	0.35±0.01^{c}
9-Nonadecene	1873.61	55.03	0±0^{b}	0±0^{a}	0.54±0.04^{a}	0.55±0.06^{a}
(S)-2,5,9,9-Tetramethyl-6,7,8,9-Tetrahydro-5H-Benzo[7]Annulene	1471.20	145.05	0.49±0.03^{b}	0.62±0.01^{a}	0.16±0.01^{c}	0.18±0^{c}
2-Isopropyl-5-Methyl-9-Methylenebicyclo[4.4.0]Dec-1-Ene	1486.89	161.09	4.81±0.35^{b}	6.62±0.33^{a}	2.25±0.24^{c}	1.14±0.05^{d}
2,5-Dimethyl-8-Propan-2-yl-1,2,8,8a-Tetrahydronaphthalene	1669.09	157.08	0.12±0.03^{a}	0.09±0^{b}	0±0^{c}	0±0^{c}
2,2-Dimethyl-3-Methylidenebicyclo[2.2.1]Heptane	945.02	93.02	0.17±0.02^{a}	0.15±0^{b}	0±0^{d}	0.12±0^{c}
Caryophyllene	1458.91	94.03	0.42±0.02^{a}	0.37±0.01^{b}	0.14±0.01^{c}	0.15±0^{c}
(1S,4S)-1,6-Dimethyl-4-Propan-2-yl-1,2,3,4-Tetrahydronaphthalene	1522.11	159.07	3.46±0.44^{a}	2.76±0.07^{b}	0.84±0.07^{c}	2.95±0.05^{b}
1-Methyl-4-(1-Methylethylidene)Cyclohexene	1083.48	93.04	4.9±0.82^{a}	3.65±0.11^{b}	0.16±0.01^{d}	2.76±0.04^{c}
3-Methyl-6-(1-Methylethylidene)Cyclohexene	1237.11	136.04	0.74±0.27^{b}	1.44±0.03^{a}	0.76±0.04^{b}	0.14±0^{c}
(4R)-4-Isopropenyl-1-Methylcyclohexene	1026.90	68.04	15.28±2.04^{a}	11.67±0.25^{b}	0.65±0.04^{d}	9.8±0.42^{c}
(2R,4aS)-4a,8-Dimethyl-2-(Prop-1-en-2-yl)-1,2,3,4,4a,5-Hexahydronaphthalene	1472.11	131.05	1.6±0.07^{b}	2.18±0.03^{a}	0.56±0.02^{c}	0.49±0.03^{d}

[2r-(2α,4aα,8aβ)]-1,2,3,4,4a,5,6,8a-Octahydro-4a,8-Dimethyl-2-(1-Methylethenyl)Naphthalene	1497.35	189.12	3.71±0.13[b]	4.26±0.21[a]	0.95±0.04[d]	1.87±0.02[c]
(1α,4aβ,8aα)-1,2,3,4,4a,5,6,8a-Octahydro-7-Methyl-4-Methylene-1-(1-Methylethyl)Naphthalene	1513.90	161.09	0.56±0.11[b]	0.47±0.01[c]	0.16±0.01[d]	0.74±0.01[a]
(1s-Cis)-1,2,3,5,6,8a-Hexahydro-4,7-Dimethyl-1-(1-Methylethyl)Naphthalene	1518.47	161.09	0.79±0.14[a]	0.65±0.03[b]	0.16±0.01[c]	0.9±0.1[a]
[1s-(1α,4aβ,8aα)]-1,2,4a,5,8,8a-Hexahydro-4,7-Dimethyl-1-(1-Methylethyl)Naphthalene	1487.56	204.15	2.23±0.04[b]	3.12±0.02[a]	0.91±0.06[c]	0.55±0.01[d]
[4ar-(4aα,7α,8aβ)]-Decahydro-4a-methyl-1-Methylene-7-(1-Methylethenyl)Naphthalene	1490.61	105.03	3.35±0.21[a]	3.15±0.14[a]	0.45±0.01[c]	1.51±0.03[b]
(4aR,8aR)-5,8a-Dimethyl-3-Propan-2-ylidene-1,2,4,4a,7,8-Hexahydronaphthalene	1543.43	161.09	1.6±0.03[b]	2.13±0.15[a]	0.52±0.02[d]	0.74±0.02[c]
Styrene	885.76	104.03	0.97±0.04[a]	0.42±0.01[b]	0.11±0.01[d]	0.25±0.01[c]
5-Ethenyl-4-Methylthiazole	1026.58	124.97	0±0[b]	0±0[b]	0.47±0.04[a]	0±0[b]
(E)-β-Ocimene	1034.25	93.04	13.79±0.83[a]	12.73±0.18[b]	0.22±0.01[c]	12.61±0.61[b]
(1R,4aR,8aR)-1,6-Dimethyl-4-(Propan-2-yl)-1,2,3,4,4a,5,6,8a-Octahydronaphthalene	1542.55	157.06	8.18±0.39[a]	7.53±0.29[b]	0.74±0.04[d]	6.93±0.39[c]
1,6-Dimethyl-4-(Propan-2-yl)Naphthalene	1617.59	185.09	0.87±0.07[b]	0.81±0.03[b]	0.19±0.01[c]	1.25±0.02[a]
(4aR,7S)-1,1,4a,7-Tetramethyl-1,2,3,4,4a,5,6,7-Octahydronaphthalene	1535.30	157.05	0.52±0.07[a]	0.5±0.01[a]	0.16±0.01[c]	0.27±0[b]
(3E,6E)-7,11-Dimethyl-3-Methylene-1,6,10-Dodecatriene	1502.97	93.03	0.81±0.02[b]	0.99±0.09[a]	0.69±0.08[c]	0.47±0.02[d]
(1R,4aR,8aR)-1,6-Dimethyl-4-(Propan-2-yl)-1,2,3,4,4a,5,6,8a-Octahydronaphthalene	1497.78	105.03	4.35±0.23[a]	4.56±0.29[a]	1.13±0.09[c]	3.39±0.08[b]
(5S)-5-Isopropyl-2-Methylcyclohexa-1,3-Diene	1003.19	93.04	5.49±0.78[a]	4.08±0.08[b]	0.18±0[c]	3.86±0.21[b]
(1E)-1-Methyl-4-(6-methylhept-5-en-2-ylidene)Cyclohex-1-Ene	1506.32	69.05	0.77±0.06[b]	0.98±0.12[a]	1.1±0.09[a]	0.36±0.06[c]
7-Methyl-3-Methyleneocta-1,6-Diene	986.70	93.04	35.59±2.77[a]	31.08±0.62[b]	0.6±0.07[c]	29.97±1.63[b]
1-Methyl-4-(1-Methylethenyl)Cyclohexene	1028.13	91.03	6.57±0.88[a]	5.16±0.14[b]	0.2±0.01[d]	4.11±0.05[c]
**Alkanes**						
[3r-(3α,3aβ,7β,8aα)]-2,3,4,7,8,8a-Hexahydro-3,6,8,8-tetramethyl-1h-3a,7-Methanoazulene	1417.95	119.04	0.25±0.01[c]	0.28±0.02[b]	0.2±0.01[d]	0.52±0.03[a]
[1ar-(1aα,4α,4aβ,7bα)]-1a,2,3,4,4a,5,6,7b-Octahydro-1,1,4,7-Tetramethyl-1H-Cyclopropa[E]Azulene	1459.71	105.03	1.25±0.06[b]	1.39±0.07[a]	0.41±0.02[d]	0.56±0.01[c]
7,7-Dimethyl-2-Methylenebicyclo[2.2.1]Heptane	1003.53	79.03	0.79±0.06[a]	0.67±0.04[b]	0.08±0.05[c]	0.7±0.04[b]
Cyclododecane	1475.00	56.04	0.73±0.02[a]	0.77±0.05[a]	0.42±0.02[c]	0.54±0.05[b]
1,1,3,5-Tetramethylcyclohexane, cis-	1047.90	125.04	0.54±0.05[a]	0±0[b]	0±0[b]	0±0[b]

1-Methylene-4-(1-Methylethenyl)Cyclohexane	1027.56	93.04	18.95±2.51^{a}	14.34±0.62^{b}	0.66±0.04^{d}	11.66±0.51^{c}
Undecylcyclohexane	1761.98	83.04	0.1±0.01^{a}	0.09±0.01^{ab}	0.11±0.01^{a}	0.08±0.01^{b}
Heneicosylcyclopentane	1862.51	69.00	0.07±0^{b}	0.08±0^{b}	0.12±0.01^{a}	0±0^{c}
Dodecane	1198.87	57.05	2.34±0.22^{b}	2.51±0.34^{b}	1.15±0.11^{c}	3.5±0.32^{a}
Heneicosane	2099.68	57.05	0±0^{b}	0±0^{b}	0.52±0.04^{a}	0±0^{b}
Hexadecane	1599.12	57.05	0.68±0.1^{b}	0.67±0.04^{b}	1.2±0.05^{a}	0.62±0.06^{b}
1,1,6-Trimethyl-1,2,3,4-Tetrahydronaphthalene	1210.24	159.06	0.14±0.01^{c}	0.71±0.04^{a}	0.21±0^{b}	0±0^{d}
1,6-Dimethyl-4-(1-Methylethyl)Naphthalene	1674.60	183.07	5.87±0.31^{a}	4.78±0.19^{b}	1.08±0.08^{c}	5.01±0.26^{b}
Nonylcyclohexane	1553.06	83.04	0.13±0.02^{a}	0.13±0.02^{a}	0.11±0^{b}	0.14±0.01^{a}
Nonadecane	1899.46	57.05	0.09±0^{c}	0±0^{c}	0.59±0.02^{b}	1.28±0.16^{a}
3-Methyl-5-Propyl-Nonane	1799.21	57.04	0.12±0^{b}	0.13±0.01^{b}	0.2±0.02^{a}	0.12±0.01^{b}
3-Methylpentadecane	1569.93	57.04	0.22±0.02^{a}	0.19±0.02^{a}	0.21±0.03^{a}	0.15±0^{b}
Tridecane	1299.02	57.05	2.68±0.59^{a}	2.56±0.33^{a}	1.54±0.28^{b}	2.61±0.08^{a}
**Others**						
(3r,5as,9ar)-2,2,5a,9-Tetramethyl-3,4,5,5a,6,7-Hexahydro-2h-3,9a-Methanobenzo[B]Oxepine	1504.73	82.01	0.5±0.04^{b}	0.63±0.01^{a}	0.16±0.01^{d}	0.35±0.01^{c}
1, 1, 5-Trimethyl-1, 2-Dihydronaphthalene	1354.55	157.05	0.89±0.01^{a}	0.76±0.02^{b}	0.17±0^{c}	0.12±0.01^{d}
Octahydro-1,4,9,9-tetramethyl-1h-3a,7-Methanoazulene	1370.86	105.05	0.67±0.21^{b}	0.85±0.02^{a}	0±0^{c}	0±0^{c}
1,1,3a,7-Tetramethyl-1a,2,3,3a,4,5,6,7b-Octahydro-1h-Cyclopropa[a]Naphthalene, [1ar-(1aα,3aα,7bα)]-	1533.33	204.14	0.65±0.03^{b}	0.99±0.05^{a}	0.2±0.02^{c}	0.18±0^{c}
1-Methylene-1h-Indene	1185.03	128.02	5.08±0.06^{b}	5.38±0.14^{a}	1.83±0.03^{d}	4.05±0.16^{c}
2,3-Dihydro-4-Methyl-1h-Indene	1146.23	117.02	0.23±0.01^{c}	0.26±0^{b}	0.11±0^{d}	0.32±0^{a}
3,4,4a,5,6,8a-Hexahydro-2h-1-Benzopyran-2,5,5,8a-Tetramethyl, (2α,4aα,8aα)-	1288.74	179.10	1.2±0.03^{a}	0.55±0.03^{b}	0±0^{c}	0±0^{c}
4-Dodecyne	1095.93	67.03	0±0^{b}	0.15±0.01^{a}	0±0^{b}	0±0^{b}
4-Isopropyl-6-Methyl-1-Methylene-1,2,3,4-Tetrahydronaphthalene	1563.40	157.05	0.32±0.06^{a}	0.31±0.04^{a}	0±0^{b}	0.36±0.02^{a}
6-Methyl-3,5,8,8a-Tetrahydro-1h-2-Benzopyran	1245.20	82.02	0.12±0.01^{c}	0.23±0.01^{b}	0±0^{d}	0.31±0^{a}
9-Methyl-S-Octahydrophenanthrene	1554.27	200.10	0.21±0.01^{b}	0.27±0.01^{a}	0.08±0.01^{c}	0.07±0^{c}
1,4-Dimethyl-7-(1-Methylethyl)Azulene	1700.79	198.08	0.13±0^{b}	0.14±0.01^{a}	0±0^{c}	0±0^{c}
Benzonitrile	1140.21	90.01	0.42±0.02^{b}	0.82±0.03^{a}	0±0^{c}	0±0^{c}
1,3-Dimethyl Naphthalene	1424.92	156.03	0.2±0.01^{b}	0.26±0^{a}	0.08±0^{d}	0.16±0.01^{c}
2-Methyl Naphthalene	1296.94	142.02	0.29±0.01^{b}	0.39±0.05^{a}	0.2±0^{d}	0.24±0.01^{c}
Methyl (z)-N-Hydroxybenzenecarboximidate	921.43	132.98	0±0^{c}	1.22±1.15^{bc}	5.26±2.86^{a}	3.17±1.19^{ab}
2,5-Dimethylpyrazine	911.78	108.03	0.98±0.08^{a}	0.97±0.01^{a}	0±0^{b}	0±0^{b}
2-Methylpyrazine	821.31	94.00	0.27±0.02^{b}	0.34±0.02^{a}	0±0^{c}	0±0^{c}

5-Ethenyltetrahydro-a,a,5-Trimethyl-(2R,5R)-rel-2-Furanmethanol	1086.09	59.01	0.22±0.05[b]	0.32±0.01[a]	0.02±0.04[d]	0.16±0.01[c]

Not: Values with different lowercase letters in superscript in the same line were significantly different at $p < 0.05$

82. Liu, T.T.; Xia, N.; Wang, Q.Z.; Chen, D.W. Identification of the non-volatile taste-active components in Crab Sauce. *Foods* **2019,** *8*, 324. https://doi.org/10.3390/foods8080324.
83. Zhou, J.; Zhang, W.; Li, G.; Xie, R.; He, J.; Hao, Y.; Li, Q.; Yu, X.; Lin, H.; Sun, Y. Analysis of flavor quality of jiulong dabaicha white tea. *Food Sci.* **2025**, *46*, 201–210.
84. Zhang, C.; Wang, X.; Liu, Y.; Wang, J.; Xie, J. Characteristics of meat flavoring prepared using hydrolyzed plant protein mix by three different heating processes. *Food Chem.* **2024**, *446*, 138853.
